# Development and Evaluation of a Cryopreserved Whole-Parasite Vaccine in a Rodent Model of Blood-Stage Malaria

**DOI:** 10.1128/mBio.02657-21

**Published:** 2021-10-19

**Authors:** Danielle I. Stanisic, Mei-Fong Ho, Reshma Nevagi, Emily Cooper, Maddison Walton, Md Tanjir Islam, Waleed M. Hussein, Mariusz Skwarczynski, Istvan Toth, Michael F. Good

**Affiliations:** a Institute for Glycomics, Griffith University, Southport, Australia; b School of Chemistry and Molecular Biosciences, The University of Queensland, St Lucia, Australia; c School of Pharmacy, The University of Queensland, St Lucia, Australia; d Institute for Molecular Bioscience, The University of Queensland, St Lucia, Australia; Max Planck Institute for Infection Biology

**Keywords:** liposome, malaria, malaria vaccine, plasmodium, whole parasite

## Abstract

Infection with malaria parasites continues to be a major global public health issue. While current control measures have enabled a significant decrease in morbidity and mortality over the last 20 years, additional tools will be required if we are to progress toward malaria parasite eradication. Malaria vaccine research has focused on the development of subunit vaccines; however, more recently, interest in whole-parasite vaccines has reignited. Whole-parasite vaccines enable the presentation of a broad repertoire of antigens to the immune system, which limits the impact of antigenic polymorphism and genetic restriction of the immune response. We previously reported that whole-parasite vaccines can be prepared using chemically attenuated parasites within intact red blood cells or using killed parasites in liposomes, although liposomes were less immunogenic than attenuated parasites. If they could be frozen or freeze-dried and be made more immunogenic, liposomal vaccines would be ideal for vaccine deployment in areas where malaria is endemic. Here, we develop and evaluate a Plasmodium yoelii liposomal vaccine with enhanced immunogenicity and efficacy due to incorporation of TLR4 agonist, 3D(6-acyl) PHAD, and mannose to target the liposome to antigen-presenting cells. Following vaccination, mice were protected, and strong cellular immune responses were induced, characterized by parasite-specific splenocyte proliferation and a mixed Th1/Th2/Th17 cytokine response. Parasite-specific antibodies were induced, predominantly of the IgG1 subclass. CD4^+^ T cells and gamma interferon were critical components of the protective immune response. This study represents an important development toward evaluation of this whole-parasite blood-stage vaccine in a phase I clinical trial.

## INTRODUCTION

Malaria is a mosquito-borne disease caused by Apicomplexan parasites of the genus, *Plasmodium*. In 2019, there were an estimated 229 million cases across 87 countries where malaria is endemic and 409,000 deaths, mainly in children <5 years of age living in Africa (1). Despite significant reductions in clinical cases and malaria-attributable deaths since 2000, progress in controlling this parasite has stalled in many countries. Insecticide-impregnated bed nets, indoor residual spraying, intermittent preventive treatment programs, and prompt diagnosis and treatment of infections have all contributed to the decline in deaths and clinical cases over the last 20 years. However, additional tools, primarily including a highly effective malaria vaccine, will be required to proceed to the eventual eradication of the malaria parasite.

The most advanced malaria vaccine, RTS,S/AS01 (Mosquirix), is a subunit vaccine based on the P. falciparum circumsporozoite protein (CSP), targeting the preerythrocytic stage of the parasite. In phase III clinical trials, following a four-dose schedule, vaccine efficacy against clinical malaria was <40% in children aged 5 to 17 months over 4 years of follow-up (2). The duration of vaccine-induced protection was short, with efficacy declining from 6 months following the final vaccine dose (2), suggesting that frequent boosters will be required to maintain protective immunity. While this limited and short-lived protection was a disappointing result for a preerythrocytic vaccine, subunit vaccine candidates targeting the blood stages of the malaria parasite have not demonstrated any efficacy when tested in the field (3–7). These subunit blood-stage vaccines all aim to induce antibodies that block merozoite invasion into red blood cells. Antigenic polymorphism, immunological nonresponsiveness, and the inability to induce and/or maintain a sufficiently high antibody response required to block invasion have all contributed to the disappointing efficacy of these subunit vaccine candidates. This has resulted in a resurgence of interest in whole-parasite vaccines, which were first modeled in ducks, monkeys, and mice in the 1940s to 1960s (8–10).

The target antigen repertoire and the mechanism/s of immunity induced by whole-parasite vaccines differ significantly from subunit vaccines. By including the entire blood-stage parasite in the vaccine, the immune system is presented with a broad repertoire of antigens, thereby limiting the impact of antigenic polymorphism and genetic restriction of the immune response to individual antigens. Furthermore, the whole parasite blood-stage vaccines tested so far all induce a cellular response, the target antigens of which are less likely to be polymorphic (11).

A number of attenuated whole-sporozoite vaccines are currently in development (reviewed in references 12 and 13); however, progress toward a whole blood-stage vaccine is much less advanced. Nevertheless, whole parasite blood-stage vaccine candidates have been assessed in rodent models of malaria. These have included killed, adjuvanted blood-stage parasites (14), genetically modified parasites (15–18), chemically attenuated blood-stage parasites (both *in vitro* [19, 20] and *in vivo* attenuation [21]), and killed blood-stage parasites formulated with liposomes (22). There has been limited clinical evaluation; however, we showed that vaccination of malaria-naive individuals with a single dose of *in vitro* chemically attenuated P. falciparum blood-stage parasites induced parasite-specific and species- and strain-transcending cellular immune responses (23).

A major challenge for attenuated blood-stage vaccines is cryopreservation. While ring-stage-infected human red cells can be cryopreserved, the low yields of viable parasites following thawing and the difficulties with preserving trophozoite and schizont stages present a significant obstacle to the development of such vaccines. One approach to overcome this is to utilize an alternative delivery system for the malaria parasites, and we have recently investigated liposomes for this purpose (22, 24). Liposomes are bilayered lipid vesicles that have flexible physiochemical and biophysical properties that make them an attractive delivery system for both drugs and for antigens for vaccine development. They are amenable to lyophilization (freeze-drying), and a lyophilized vaccine would have significant advantages for deployment in areas where malaria is endemic.

Here, we demonstrate that an adjuvanted and targeted liposomal vaccine containing P. yoelii 17X blood-stage parasites, which can be lyophilized without losing potency, can induce strain-transcending immune responses that contribute significantly to protection from infection. We also define the immune correlates of protection.

## RESULTS

### Optimizing vaccine components and delivery.

**FIG 1:**
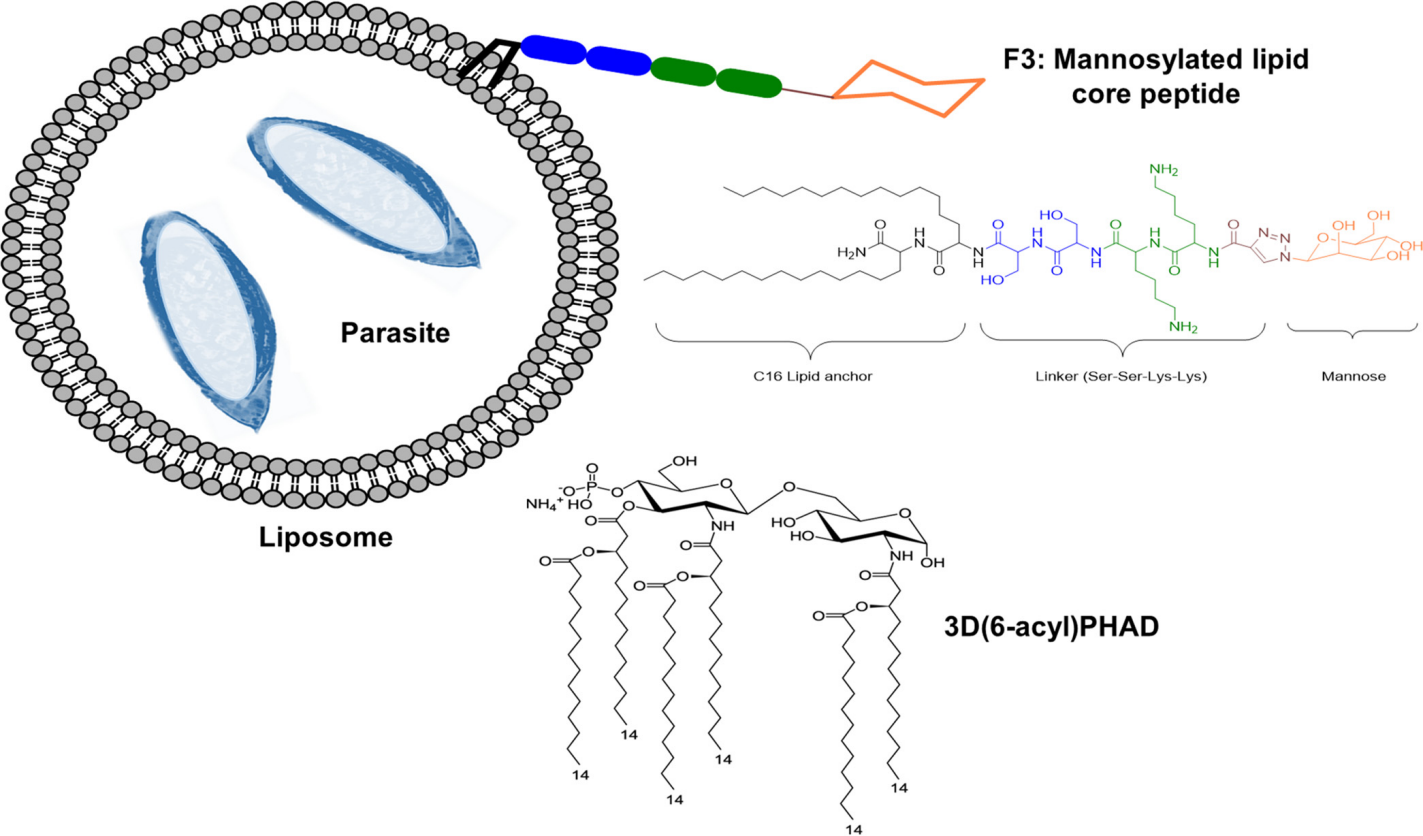
Schematic of the P. yoelii 17X liposomal vaccine. The liposomal vaccine was prepared using thin-film hydration with a mannosylated lipid core peptide (“F3”) as described previously (22) and 3D(6-acyl) PHAD. The liposomes contained pRBCs killed by freeze-thawing. (Adapted from Al-Nazal et al. [42].)

**FIG 2:**
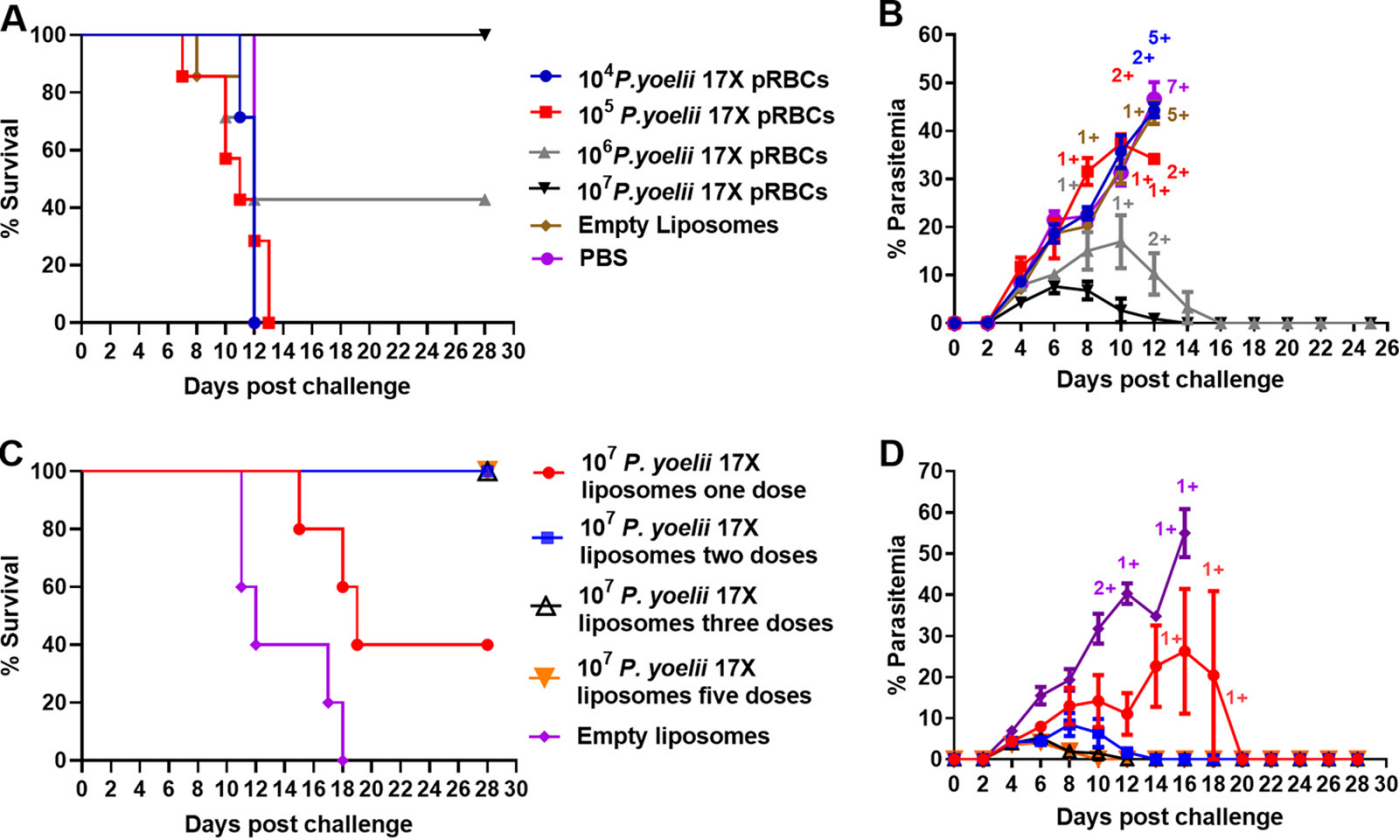
Comparison of the protective efficacy of a P. yoelii 17X F3 + PHAD liposomal blood-stage vaccine in relation to parasite dose and the number of vaccine doses. For panels A and B, groups of BALB/c mice (*n* = 7/group) received three subcutaneous immunizations of an F3 + PHAD liposome vaccine containing different quantities of P. yoelii 17X pRBCs. For panels C and D, the dosing regimen was varied with groups of BALB/c mice (*n* = 5/group) receiving one, two, three, or five doses of an F3 + PHAD liposome vaccine containing 10^7^ P. yoelii 17X pRBCs. All mice were challenged i.v. 1 month after the final vaccine dose with 10^5^ P. yoelii 17X pRBCs. (A and C) Survival of mice after vaccination and blood-stage challenge. Mice were monitored daily following challenge and euthanized if indicated by their clinical score or a weight loss of >15% postchallenge. (B and D) Parasitemias were monitored by blood films every second day following challenge. +, Mice that were euthanized based on their clinical score. The data are presented as means ± the SEM.

We previously showed that a whole parasite blood-stage vaccine, consisting of 10^6^ P. yoelii or P. chabaudi parasitized red blood cells (pRBCs) formulated with liposomes modified with the mannosylated lipid core peptide, F3, controlled parasite burden following homologous blood-stage parasite challenge (22). In these experiments, all vaccinated mice survived, but some experienced high parasitemias. We sought to determine whether increasing the antigen dose in the vaccine and adding the synthetic form of the TLR-4 agonist MPLA, 3D(6-acyl) PHAD (25) (Fig. 1), would improve vaccine efficacy (Fig. 2A and B). We observed that the protective efficacy of a P. yoelii 17X vaccine in BALB/c mice was parasite dose dependent, with 100% survival observed in the group of mice immunized with the vaccine containing 10^7^ P. yoelii 17X pRBCs, 40% survival observed when the vaccine contained 10^6^ pRBCs, and no survival when the vaccine contained 10^4^ or 10^5^ pRBCs (Fig. 2A). Mice vaccinated with the highest parasite dose also had the lowest peak parasitemia (7.65% ± 1.422%) of all groups (Fig. 2B). Liposomes containing 10^7^ pRBCs, F3, and PHAD were selected for further experiments. In BALB/c mice, we observed that two, three, or five doses of vaccine gave optimal protection in terms of survival and lower peak parasitemia, compared to one dose of vaccine (Fig. 2C and D). Area under the curve (AUC) analysis showed that three doses was superior to two doses, but this difference was not statistically significant. Five doses of the vaccine were not better than three. A vaccination regimen consisting of three doses containing 10^7^ P. yoelii 17X pRBCs was selected for further experiments.

**FIG 3:**
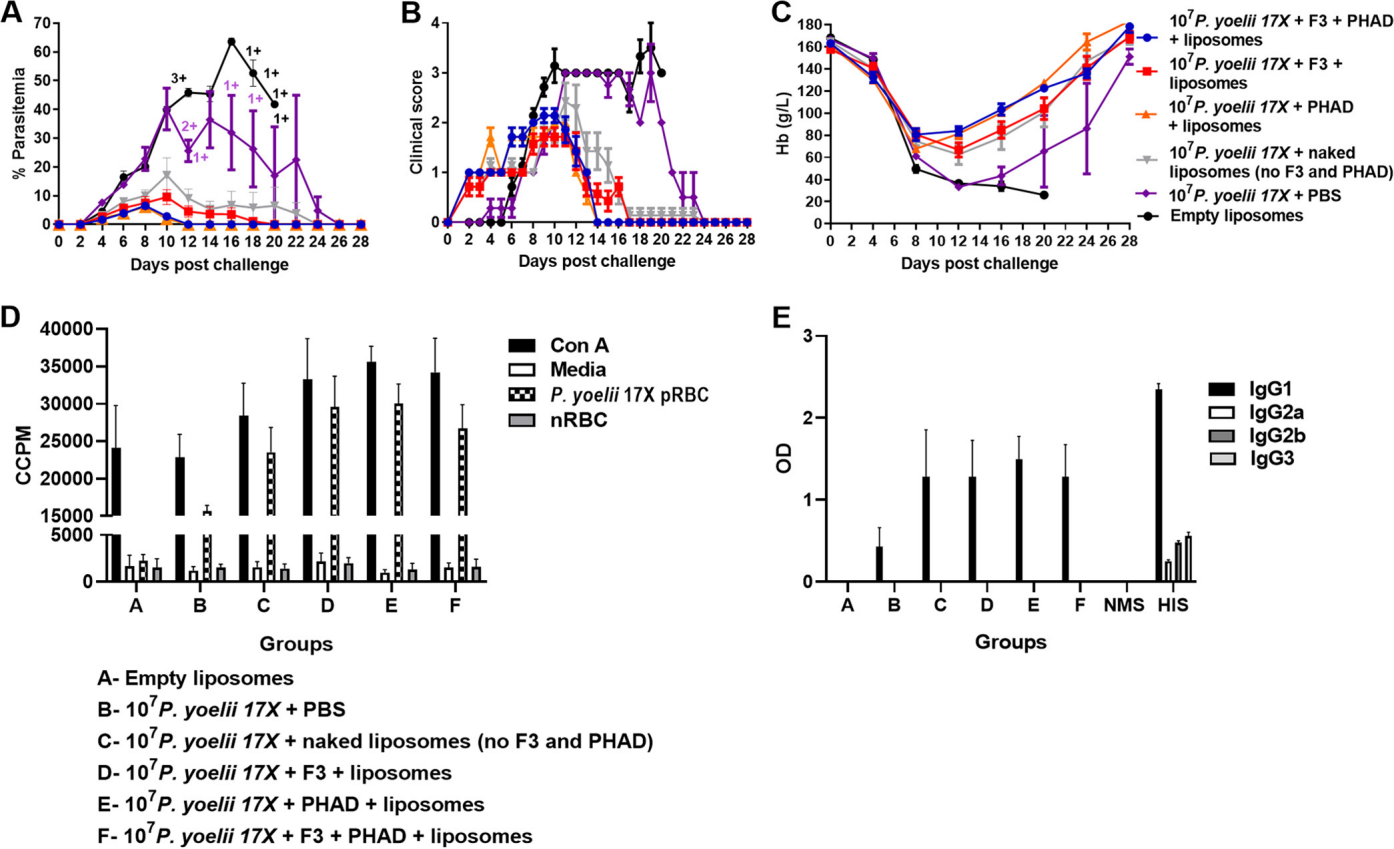
Effect of targeting ligands on the immunogenicity and protective efficacy of the 10^7^ P. yoelii 17X F3 + PHAD liposomal blood-stage vaccine. Groups of BALB/c mice (*n* = 7 mice/group) were immunized with three doses of liposomal vaccine formulations containing 10^7^ P. yoelii 17X pRBCs and different combinations of the targeting ligands F3 and PHAD. Mice were challenged 1 month after the final vaccine dose with 10^5^ P. yoelii 17X pRBCs. Control mice were immunized with empty liposomes. (A) Parasitemias were monitored by blood films every second day after challenge. (B) Mice were monitored daily after challenge and euthanized if indicated by their clinical score or a weight loss of >15% postchallenge. (C) Hemoglobin levels were measured using a Hemocue201^+^ Analyser. (D) Prechallenge splenocyte proliferative responses of vaccinated and control mice in response to fresh P. yoelii 17X pRBCs (*n* = 3/group). Proliferation was estimated by ^3^[H]thymidine incorporation and measured as corrected counts per minute (CCPM). Splenocytes from each mouse were tested with each stimulant in triplicate. (E) Prechallenge parasite-specific IgG subclass responses in vaccinated and control mice (*n* = 7/group). Antibody to crude whole P. yoelii 17X parasite antigen was measured by ELISA. Serum was tested in duplicate at 1:100; samples from naive mice (NMS; normal mouse serum) and hyperimmune mice (HIS; hyperimmune serum) were included as negative and positive controls, respectively. The results are expressed as the optical density (OD) at 450 nM. The data are presented as means ± the SEM. +, Mice that were euthanized based on their clinical score.

We next examined the contribution of the different adjuvant components in the vaccine to both immunogenicity and efficacy in BALB/c mice (Fig. 3). Mice vaccinated with empty liposomes or parasite antigen in saline had the highest parasitemias, highest clinical scores, and lowest hemoglobin levels postchallenge (Fig. 3A to C). All groups that were vaccinated with formulations containing parasite antigen and liposomes had 100% survival (Fig. 3A). Groups vaccinated with liposomes containing PHAD (either PHAD alone or PHAD and F3) had lower peak parasitemias than groups vaccinated with liposomes not containing PHAD (Fig. 3A); these differences were not significant. However, mice vaccinated with liposomes containing PHAD cleared patent parasitemias more quickly than mice vaccinated with liposomes containing F3 only (*P* ≤ 0.02 for both comparisons). A trend was observed for better hemoglobin levels in mice that received liposomes containing PHAD, but these differences were not significant. We studied the humoral and cellular immune responses in these vaccinated mice. All liposomal formulations containing P. yoelii 17X antigen induced significantly higher parasite-specific splenocyte proliferative responses than empty liposomes or parasite antigen in saline (Fig. 3D) (*P* ≤ 0.0016 for all comparisons). All vaccine formulations that contained P. yoelii 17X antigen induced significantly higher levels of IL-6, IL-4, IL-2, and MCP-1 than empty liposomes (*P* < 0.05) (Table 1). However, only vaccine formulations containing PHAD induced significant amounts of IFN-γ and only formulations containing F3 induced significant amounts of IL-10 (*P* < 0.05). All formulations containing F3 and/or PHAD induced tumor necrosis factor (TNF), and only formulations containing both F3 and PHAD induced IL-17A that was significantly higher than empty liposomes (*P* < 0.05). The levels of IgG1 induced by all liposomal formulations were significantly higher than levels induced by parasite antigens in saline (*P* ≤ 0.0031 for all comparisons) (Fig. 3E). IgG1 levels did not differ significantly between mice vaccinated with any of the liposomal formulations containing parasite antigen (Fig. 3E). IgG1 was the only IgG subclass that was detected, with IgG2a, IgG2b, and IgG3 only detected in the hyperimmune serum (HIS) of mice that had undergone multiple infection/drug cure cycles.

**TABLE 1:** Parasite-specific cytokines and chemokines produced by splenocytes from mice vaccinated with different P. yoelii 17X liposomal vaccine formulations^*a*^

Formulation	Mean pg/ml ± SEM								
	IL-10	IL-17A	TNF	IFN-γ	IL-6	IL-4	IL-2	IL-12p70	MCP-1
Empty liposomes	0 ± 0	0 ± 0	9.68 ± 9.51	0 ± 0	1.87 ± 1.70	0.49 ± .049	0 ± 0	14.59 ± 7.68	37.17 ± 29.81
10^7^ Py + PBS	12.55 ± 4.55	0 ± 0	25.51 ± 2.82	5.64 ± 2.30	**38.74 ± 4.97**	**12.77 ± 1.76**	**118.30 ± 7.12**	12.34 ± 9.14	**1,384.85 ± 275.85**
10^7^ Py + naked liposomes^*b*^	25.21 ± 9.43	24.36 ± 19.72	62.36 ± 23.64	137.10 ± 112.42	**94.11 ± 20.27**	**20.82 ± 3.54**	**207.70 ± 46.59**	28.52 ± 28.52	**1,463.99 ± 316.11**
10^7^ Py + F3 + liposomes	**53.93 ± 16.26**	148.78 ± 61.03	**195.90 ± 65.95**	415.84 ± 215.88	**211.43 ± 42.18**	**34.49 ± 5.06**	**496.19 ± 114.15**	5.91 ± 5.91	**2,429.14 ± 557.63**
10^7^ Py + PHAD + liposomes	41.80 ± 27.57	304.27 ± 127.11	**368.81 ± 90.66**	**1,173.27 ± 509.85**	**245.38 ± 77.17**	**41.04 ± 6.64**	**526.81 ± 101.60**	9.56 ± 5.08	**1,274.49 ± 308.79**
10^7^ Py + F3 + PHAD + liposomes	**23.83 ± 9.47**	**161.03 ± 54.89**	**303.13 ± 14.07**	**1,830.55 ± 89.64**	**220.67 ± 47.48**	**48.91 ± 11.3**	**485.54 ± 20.45**	2.40 ± 2.22	**1,261.51 ± 163.08**
10^7^ Py + F3 + PHAD liposomes (lyophilized)^*c*^	**23.75 ± 8.05**	**402.78 ± 52.73** ^*d*^	**659.61 ± 100.32** ^*d*^	**3,636.62 ± 472.27** ^*d*^	**335.15 ± 39.31**	**79.33 ± 8.73**	**666.09 ± 89.49**	4.43 ± 2.22	**1,040.86 ± 159.06**

a Parasite-specific cytokine and chemokine production in the culture supernatants of splenocyte proliferation assays that were collected after 54 h and used in cytokine bead arrays (*n* = 3/group). The values reflect P. yoelii 17X pRBC responses that have been normalized by subtracting the cytokine responses to normal red blood cells (nRBCs). The supernatants were pooled from triplicate wells for three mice/group. Abbreviations: IFN, interferon; IL, interleukin; MCP, monocyte chemoattractant protein; Py, P. yoelii 17X; TNF, tumor necrosis factor. Boldface values are significantly different from the empty liposome group (*P* < 0.05).

b Naked liposomes have no F3 or PHAD included in the formulation.

c Only this vaccine formulation was lyophilized. All other vaccine formulations were freshly prepared.

d Significantly different from the fresh 10^7^ Py + F3 + PHAD + liposome group (*P* < 0.05).

**FIG 4:**
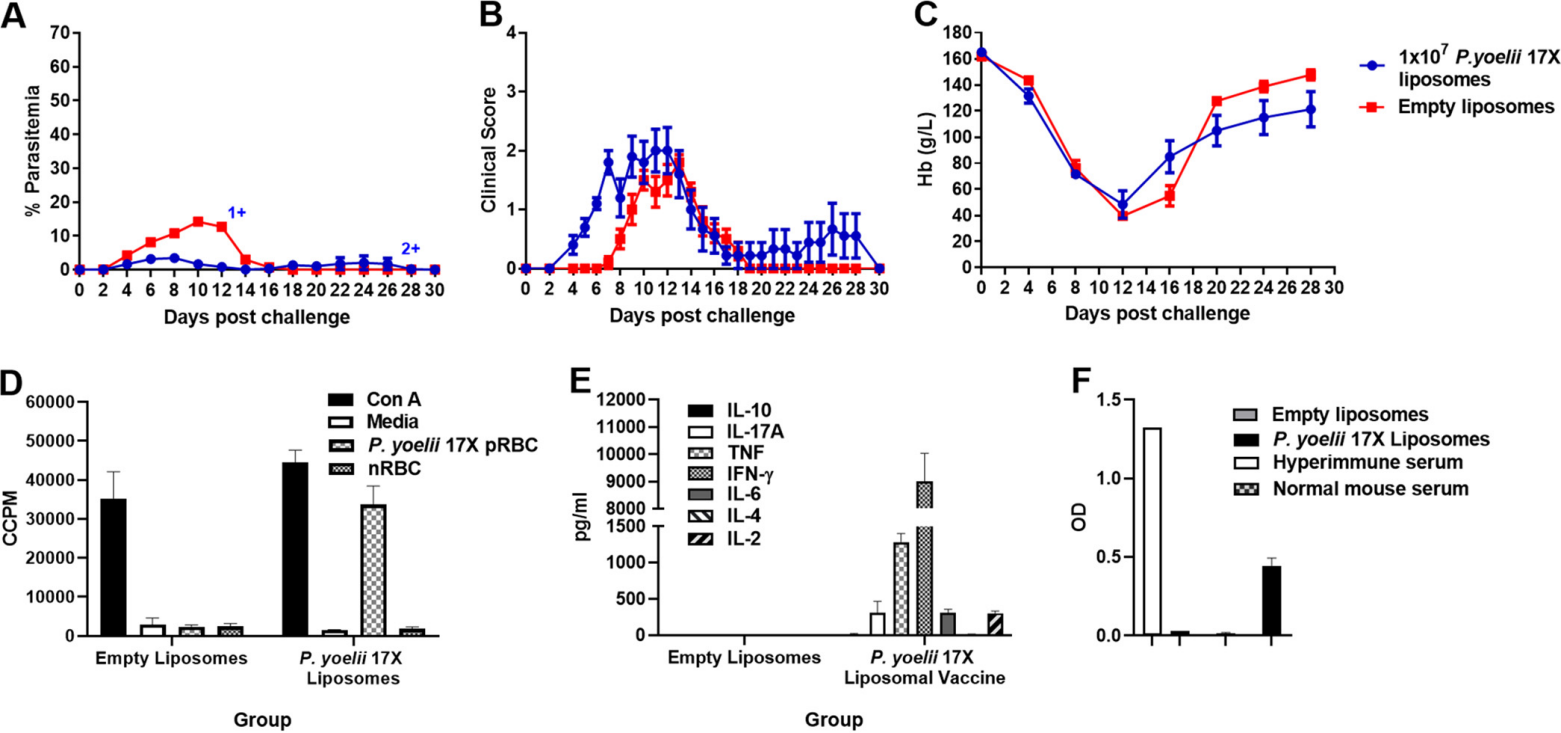
Immunogenicity and protective efficacy of a 10^7^ P. yoelii 17X F3 + PHAD liposomal blood-stage vaccine in outbred Swiss mice. Groups of Swiss mice (*n* = 10/group) were immunized with three doses of the F3 + PHAD liposome vaccine containing 10^7^ P. yoelii 17X pRBCs and challenged 1 month after the final vaccine dose with 10^5^ P. yoelii 17X pRBCs. Control mice were immunized with empty liposomes. (A) Parasitemias were monitored by blood films every second day after challenge. (B) Mice were monitored daily after challenge and euthanized if indicated by their clinical score or a weight loss of >15% postchallenge. (C) Hemoglobin levels were measured using a Hemocue201^+^ Analyser. (D) Prechallenge splenocyte proliferative responses of vaccinated and control mice in response to fresh P. yoelii 17X pRBCs (*n* = 3/group). Proliferation was estimated by ^3^[H]thymidine incorporation and measured as corrected counts per minute (CCPM). Splenocytes from each mouse were tested in triplicate with each stimulant. (E) Parasite-specific cytokine production in the culture supernatants of the splenocyte proliferation assay that were collected after 54 h and used in cytokine bead arrays (*n* = 3/group). The values reflect P. yoelii 17X pRBC responses that have been normalized by subtracting the cytokine responses to normal red blood cells (nRBCs). The supernatants were pooled from triplicate wells for three mice/group. (F) Prechallenge parasite-specific IgG responses in vaccinated and control mice (*n* = 10/group). Antibody to crude whole P. yoelii 17X parasite antigen was measured by ELISA. Serum was tested in duplicate at 1:100; samples from naive mice (normal mouse serum) and hyperimmune mice were included as negative and positive controls, respectively. The results are expressed as the optical density (OD) at 450 nM. The data are presented as means ± the SEM. +, Mice that were euthanized based on their clinical score. Abbreviations: ConA, concanavalin A; IFN, interferon; IL, interleukin; MCP, monocyte chemoattractant protein; TNF, tumor necrosis factor.

We examined the immunogenicity and efficacy of the vaccine in outbred Swiss mice, which may better represent genetically diverse humans, and observed similar outcomes to those seen in BALB/c mice in terms of reduced peak parasitemia (*P* < 0.0001) (Fig. 4A) and splenocyte proliferative (Fig. 4D) and cytokine responses (Fig. 4E). The levels of parasite-specific IgG were significantly higher in mice that received the P. yoelii 17X liposomes compared to the empty liposomes (*P* < 0.0001) (Fig. 4F). However, after challenge, 3/10 vaccinated mice required euthanasia due to clinical score (one mouse) or to >15% weight loss postchallenge (two mice) despite having low parasitemias at the time (Fig. 4A). Peak clinical scores and the lowest hemoglobin levels were not significantly different between vaccinated and control mice (Fig. 4B and C) (*P* > 0.05). It is possible that the mortality may reflect the fact that the vaccine is produced from the infected blood of outbred Swiss mice, which contains white blood cells that have genetically diverse MHC antigenic expression that when administered to other outbred mice may lead to a deleterious inflammatory response. This would not be seen in BALB/c mice, which are an inbred strain. This is supported by the observation that the IFN-γ response was approximately five times higher in the vaccinated Swiss mice compared to the BALB/c mice (9,002 pg/ml ± 1,031.95 pg/ml) (Fig. 4E) and 1,830.55 pg/ml ± 89.64 pg/ml (Table 1), respectively).

### Heterologous protective immunity.

**FIG 5:**
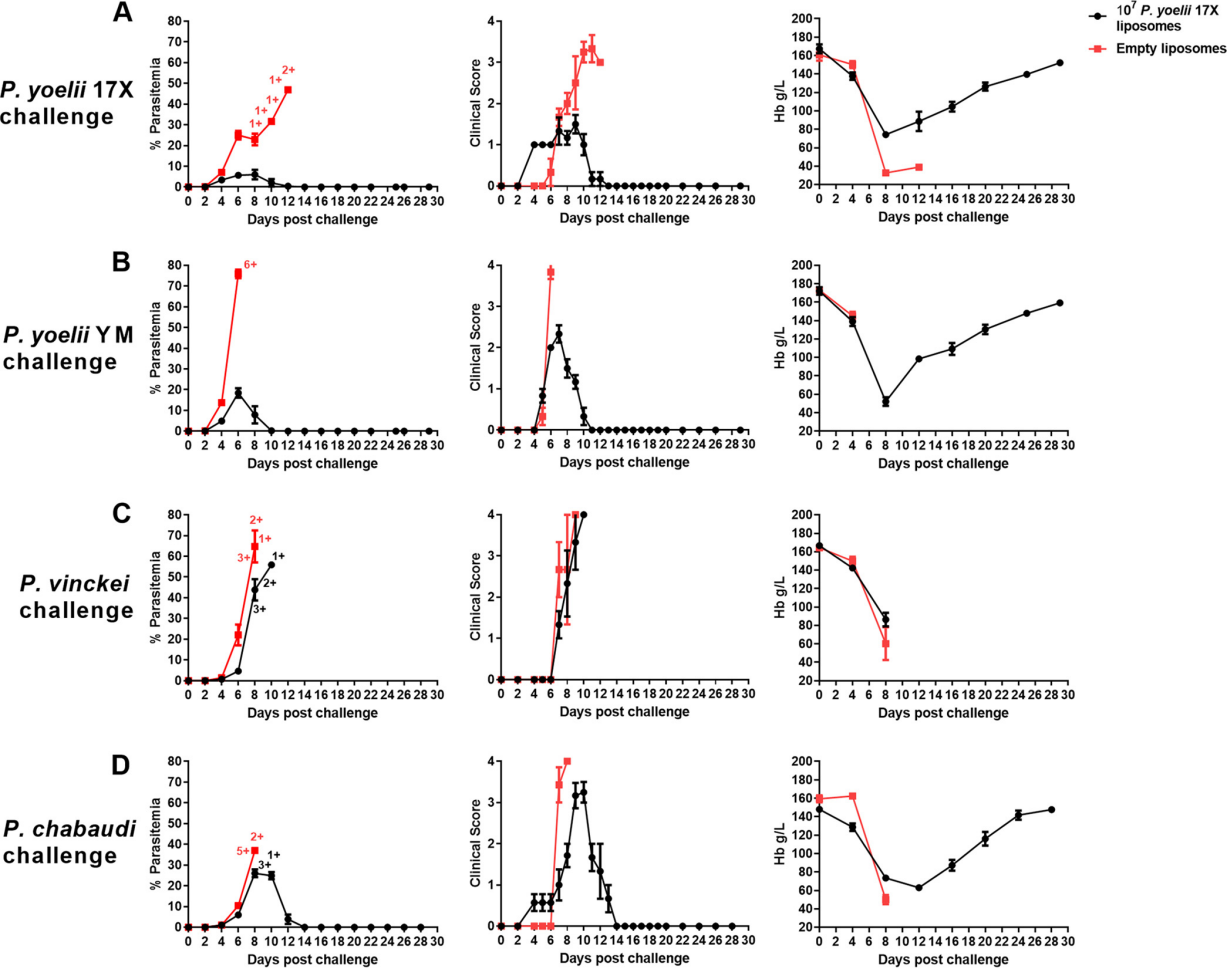
Heterologous protective efficacy of a 10^7^ P. yoelii 17X F3 + PHAD liposomal blood-stage vaccine. Groups of BALB/c mice were immunized with three doses of the F3 + PHAD liposome vaccine containing 10^7^ P. yoelii 17X pRBCs and challenged 1 month after the final vaccine dose with 10^5^ P. yoelii 17X pRBCs (*n* = 6/group) (A), P. yoelii YM pRBCs (*n* = 6/group) (B), *P. vinckei* pRBCs (*n* = 6/group) (C), or P. chabaudi pRBCs (*n* = 7/group) (D). Control mice were immunized with empty liposomes. Parasitemias (first column) were monitored by blood films every second day after challenge. Mice were monitored daily for their clinical scores (second column) after challenge and euthanized if indicated by their clinical score or a weight loss of >15% postchallenge. Hemoglobin levels (third column) were measured using a Hemocue201^+^ Analyser. +, Mice that were euthanized based on their clinical score or a weight loss of >15% postchallenge. The data are presented as means ± the SEM.

In BALB/c mice we compared vaccine efficacy after challenge with a homologous parasite (P. yoelii 17X), a heterologous strain parasite (P. yoelii YM), and a heterologous parasite species (P. chabaudi, *P. vinckei*) (Fig. 5). We measured parasite density and clinical outcomes (clinical score, hemoglobin). Control of parasite burden and clinical scores were better when vaccinated mice were challenged with homologous parasites, compared to all other parasites. However, all mice challenged with the heterologous strain, P. yoelii YM, survived and were able to clear parasites microscopically within 10 days. There was no protection against *P. vinckei* and limited protection against P. chabaudi.

### CD4^+^ T cells are critical mediators of vaccine-induced immunity.

**FIG 6:**
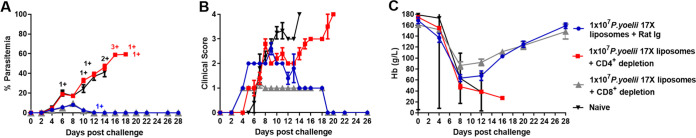
Role of CD4^+^ and CD8^+^ T cells in protective immunity induced by a 10^7^ P. yoelii 17X F3 + PHAD liposomal blood-stage vaccine. Groups of BALB/c mice (*n* = 5/group) were immunized with three doses of the F3 + PHAD liposome vaccine containing 10^7^ P. yoelii 17X pRBCs and challenged 1 month after the final vaccine dose with 10^5^ P. yoelii 17X pRBCs. Vaccinated mice received injections of rat Ig, anti-CD4^+^, or anti-CD8^+^ antibodies on days −2, −1, 4, and 8 relative to the challenge on day 0. A naive control group that only received the challenge was also included. (A) Parasitemias were monitored by blood films every second day after challenge. (B) Mice were monitored daily after challenge and euthanized if indicated by their clinical score or a weight loss of >15% postchallenge. (C) Hemoglobin levels were measured using a Hemocue201^+^ Analyser. The data are presented as means ± the SEM. +, Mice that were euthanized based on their clinical score or a weight loss of >15% postchallenge.

The strong cytokine responses produced by splenocytes from vaccinated mice was a possible indicator of an important role for CD4^+^ T cells in vaccine-induced immunity. To test this, we undertook T cell depletion studies. Vaccinated BALB/c mice were given injections of rat immunoglobulin (Ig) (control), anti-CD4^+^ or anti-CD8^+^ antibodies on days −2, −1, 4, and 8 relative to the challenge on day 0. At the time of challenge, >99% depletion of CD4^+^ T cells and >93% depletion of CD8^+^ T cells were observed (data not shown). After depletion, only vaccinated mice that received CD8^+^ T-cell-depleting antibodies or rat Ig survived challenge, while mice injected with CD4^+^ T-cell-depleting antibodies demonstrated a complete inability to control parasitemia, and all succumbed to infection (Fig. 6A). Thus, CD4^+^ but not CD8^+^ T cells are critical for immunity. Despite having very similar parasitemias, vaccinated CD8^+^ T-cell-depleted mice had significantly lower peak clinical scores (1.2 ± 0.2 versus 2.6 ± 0.24; *P* = 0.004) (Fig. 6B) (*P* = 0.004) and were better able to maintain hemoglobin levels (lowest level 86.6 ± 6.705 g/liter versus 63.4 ± 6.35 g/liter; *P* = 0.036) (Fig. 6C) than the vaccinated mice that received rat Ig, suggesting that vaccine-induced CD8^+^ T cells contributed to pathology.

**FIG 7:**
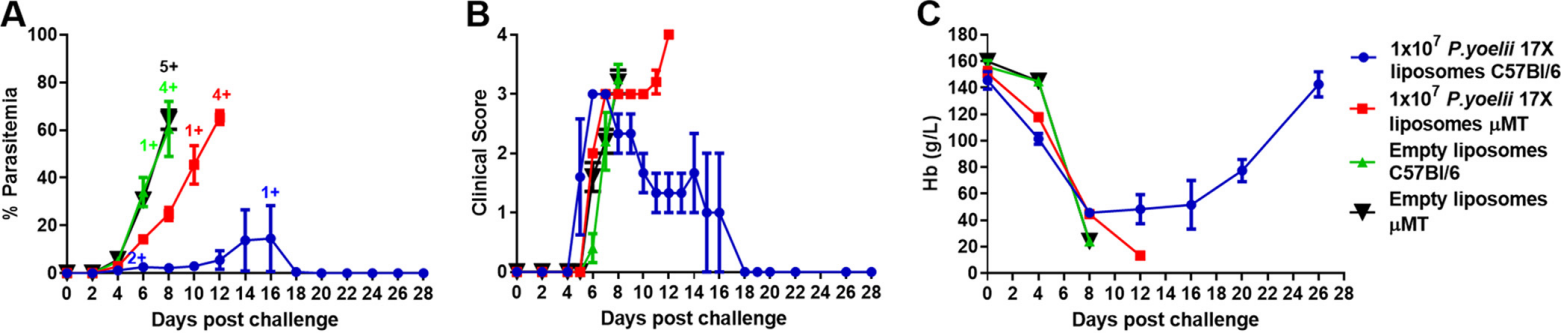
Role of B cells in protective immunity induced by a 10^7^ P. yoelii 17X F3 + PHAD liposomal blood-stage vaccine. Groups of immunocompetent C57BL/6 and immunodeficient μMT mice (deficient in mature B cells) (*n* = 5/group) were immunized with three doses of the F3 + PHAD liposome vaccine containing 10^7^ P. yoelii 17X pRBCs and challenged 1 month after the final vaccine dose with 10^5^ P. yoelii 17X pRBCs (*n* = 5/group). Control mice were immunized with empty liposomes. (A) Parasitemias were monitored by blood films every second day after challenge. (B) Mice were monitored daily after challenge and euthanized if indicated by their clinical score or a weight loss of >15% postchallenge. (C) Hemoglobin levels were measured using a Hemocue201^+^ Analyser. The data are presented as means ± the SEM. +, Mice that were euthanized based on their clinical score or a weight loss of >15% postchallenge.

To investigate the role of B cells in vaccine-induced immunity, immunocompetent C57BL/6 mice and immunodeficient μMT mice were vaccinated and challenged. For this experiment, C57BL/6 mice were used since they are the wild-type counterpart of μMT mice. B-cell development is abnormal in μMT mice, and they lack mature B cells and antibodies (26). However, these mice also have an altered T-cell phenotype following infection, which may complicate interpretation of data (27). After vaccination, prechallenge sera from C57BL/6 mice contained parasite-specific antibodies, whereas antibodies were not detected in the μMT mice (data not shown). After challenge, the μMT mice that received the P. yoelii 17X liposomes and control mice succumbed to infection, although the parasite growth rate was reduced in the vaccinated μMT mice compared to the control mice (Fig. 7A). All vaccinated mice developed a high clinical score which was independent of the peripheral blood parasitemia (Fig. 7B). These data suggest that B cells are a key component of the protective immune response induced by the P. yoelii 17X liposomal vaccine. However, passive transfer of serum from vaccinated BALB/c mice to naive BALB/c mice (0.5 ml administered to naive recipients on days −1, 0, and 1 relative to the challenge on day 0) did not transfer any protection (see Fig. S1) suggesting that the lack of other factors in μMT mice, apart from B cells, or other alterations in the immune response, contributed to their inability to be protected by vaccination.

10.1128/mBio.02657-21.1 FIG S1 Role of antibodies induced by a 10^7^ P. yoelii 17X F3 + PHAD liposomal blood-stage vaccine. Groups of naive BALB/c mice (five mice/group) were injected intraperitoneally with either serum from naive BALB/c mice (normal mouse serum) or serum from BALB/c mice immunized with three doses of the F3 + PHAD liposomal vaccine (vaccinated serum) containing 10^7^ P. yoelii 17X pRBCs. The naive recipient mice received 0.5ml of serum on days −1, 0, and 1 relative to i.v. challenge with 10^5^ P. yoelii 17X pRBCs on day 0. A naive control group that only received the challenge was also included. Parasitemias were monitored by blood films every second day following challenge. The data are presented as means ± the SEM. +, Mice that were euthanized based on their clinical score or a weight loss of >15% postchallenge. Download FIG S1, TIF file, 0.2 MB. Copyright © 2021 Stanisic et al. 2021 Stanisic et al. https://creativecommons.org/licenses/by/4.0/ This content is distributed under the terms of the Creative Commons Attribution 4.0 International license.

### Macrophages and neutrophils are not required for vaccine-induced immunity.

CD4^+^ T cells are not known to directly kill the malaria parasite but can indirectly exert antiparasite activity through the production of key cytokines such as IFN-γ and TNF, which are essential mediators of the parasite-specific adaptive immune response (reviewed in reference 28). These cytokines can subsequently activate other cells of the immune system, e.g., neutrophils and macrophages, which may directly kill the malaria parasite through phagocytosis and the production of cytotoxic substances. To determine the role of neutrophils and macrophages in vaccine-induced immunity, we performed depletion studies.

**FIG 8:**
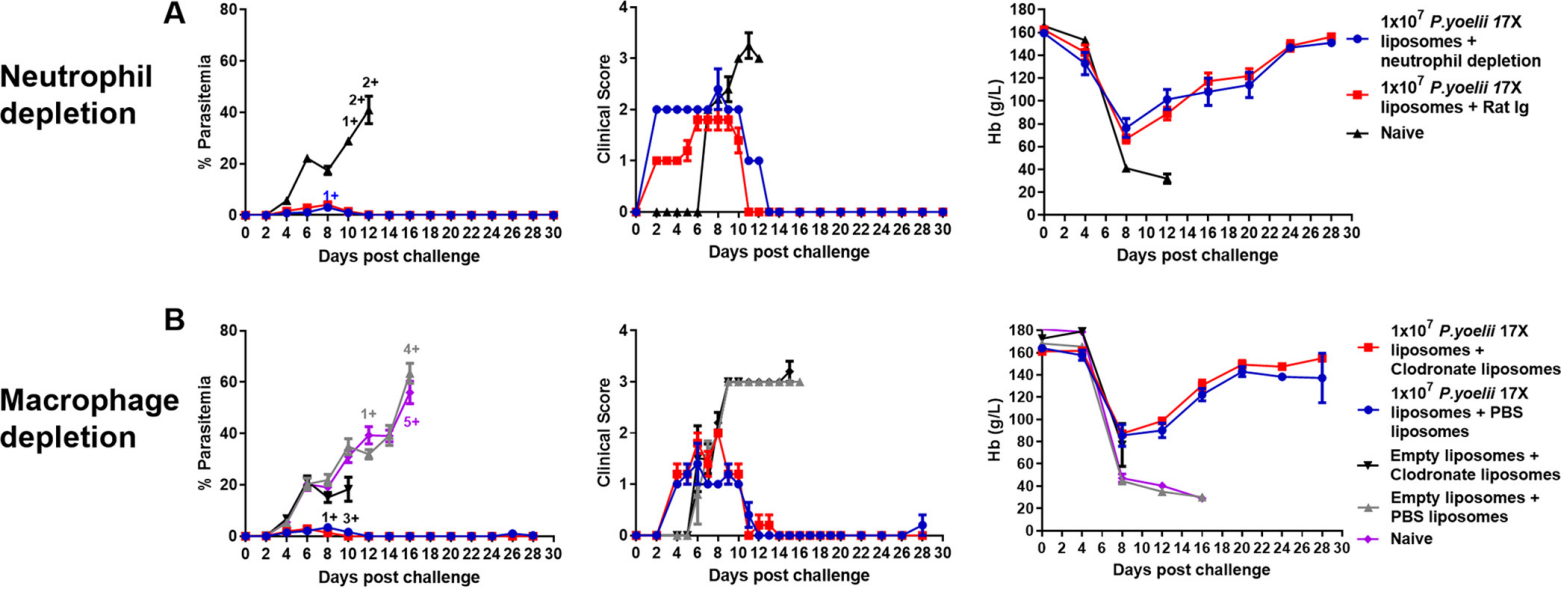
Role of macrophages and neutrophils in protective immunity induced by a 10^7^ P. yoelii 17X F3 + PHAD liposomal blood-stage vaccine. Groups of BALB/c mice (*n* = 5 mice/group) were immunized with three doses of the F3 + PHAD liposome vaccine containing 10^7^ P. yoelii 17X pRBCs and challenged 1 month after the final vaccine dose with 10^5^ P. yoelii 17X pRBCs. A naive control group that only received the challenge was also included. (A) To examine the role of neutrophils in protective immunity, vaccinated mice received injections of rat Ig or anti-Ly6G antibodies on days −4, 1, 2, 5, 8, 11, and 14 relative to the challenge on day 0. (B) To examine the role of macrophages in protective immunity, vaccinated mice received 100 to 200 μl of a liposome suspension containing clodronate (5 mg/ml) or PBS on days −1 and 7 relative to the challenge on day 0. Parasitemias (first column) were monitored by blood films every second day following challenge. Mice were monitored daily for their clinical scores (second column) after challenge and euthanized if indicated by their clinical score or a weight loss of >15% postchallenge. Hemoglobin levels (third column) were measured using a Hemocue201^+^ Analyser. +, Mice that were euthanized based on their clinical score or a weight loss of >15% postchallenge. The data are presented as means ± the SEM. All groups had five mice except for the group that was vaccinated with empty liposomes and received treatment with the clodronate liposome group, which had four mice.

To examine the role of neutrophils, vaccinated BALB/c mice were given injections of rat Ig or anti-Ly6G antibody on days −4, 1, 2, 5, 8, 11, and 14, relative to challenge on day 0. At the time of challenge, >99% depletion of neutrophils was observed (data not shown). Naive mice quickly succumbed to the infection (Fig. 8A). Vaccinated mice that received either rat Ig or the anti-Ly6G antibody developed low-level parasitemias that were not significantly different between the two groups of mice (*P* > 0.05) (Fig. 8A). Similarly, there was no significant difference in the mean peak clinical scores or hemoglobin levels (Fig. 8A).

To determine the role of macrophages, vaccinated BALB/c mice were given injections of liposomes containing either phosphate-buffered saline (PBS) or clodronate on days −1 and 7 relative to the challenge on day 0. Macrophages ingest the liposomes and, since clodronate is toxic, the clodronate liposomes effectively deplete the macrophages (29). While the naive mice and mice vaccinated with empty liposomes succumbed to challenge, the groups of macrophage-depleted and -nondepleted vaccinated mice both survived challenge infection, developing low-level parasitemias that were not significantly different between the two groups (*P* > 0.05) (Fig. 8B). There were no significant differences between hemoglobin levels or mean peak clinical scores (Fig. 8B). These data indicate that vaccine-induced immunity is not dependent on either neutrophils or macrophages.

### Role of cytokines in vaccine-induced immunity.

**FIG 9:**
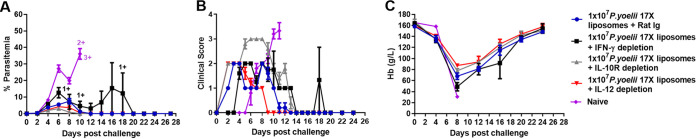
Role of key proinflammatory and anti-inflammatory cytokines in protective immunity induced by a 10^7^ P. yoelii 17X F3 + PHAD liposomal blood-stage vaccine. Groups of BALB/c mice (five mice/group) were immunized with three doses of the F3 + PHAD liposome vaccine containing 10^7^ P. yoelii 17X pRBCs and challenged 1 month after the final vaccine dose with 10^5^ P. yoelii 17X pRBCs. A naive control group that only received the challenge was also included. To examine the role of key proinflammatory and anti-inflammatory cytokines, mice received injections of rat Ig, anti-IFN-γ, anti-IL-12, or anti-IL-10R antibodies on days −1, 0, and 1 relative to the challenge on day 0. (A) Parasitemias were monitored by blood films every second day after challenge. (B) Mice were monitored daily after challenge and euthanized if indicated by their clinical score or a weight loss of >15% postchallenge. (C) Hemoglobin levels were measured using a Hemocue201^+^ Analyser. The data are presented as means ± the SEM. +, Mice that were euthanized based on their clinical score or a weight loss of >15% postchallenge.

Inflammatory cytokines have been shown to directly kill intraerythrocytic parasites in a process known as “crisis” (30–33). We examined the role of key proinflammatory and anti-inflammatory cytokines following the challenge of vaccinated mice. BALB/c mice were given injections of rat Ig (control), anti-IFN-γ, anti-IL-12p40, or anti-IL-10R antibodies on days −1, 0, and 1 relative to the challenge on day 0. Mice that were treated with anti-IFN-γ antibodies had a higher peak parasitemia (Fig. 9A) and a lower hemoglobin level (Fig. 9C) than the mice treated with rat Ig. In addition, the depletion of IFN-γ impacted survival; this was the only vaccinated group that had <100% survival, with 3/5 mice succumbing to infection. The mean peak parasitemia of the mice that were treated with anti-IL-10R antibody was significantly lower than in mice treated with rat Ig (3.02% ± 0.30% versus 7.54% ± 1.66%) (*P* = 0.028) (Fig. 9A). In agreement with the anti-inflammatory role of IL-10, mice treated with anti-IL-10R antibodies had a significantly higher peak clinical score than all other vaccinated, treated groups after challenge (Fig. 9B) (*P*< 0.001). Mice treated with anti-IL-12p40 antibodies had similar parasitemias, clinical scores, and hemoglobin levels to mice treated with rat Ig (Fig. 9).

In a follow-up experiment, we examined the impact of depleting both IFN-γ and TNF by administering injections of rat Ig (control), anti-IFN-γ, anti-TNF, or both anti-IFN-γ and anti-TNF on days −1, 1, 3, and 5 relative to the challenge on day 0. BALB/c mice that were treated with anti-IFN-γ, anti-TNF, or both of these anti-cytokine antibodies had higher peak parasitemias (see Fig. S2) than the mice treated with rat Ig, while depletion of IFN-γ or both IFN-γ and TNF impacted survival, with 3/5 and 4/5 mice succumbing to infection, respectively. Together, these data suggest that after vaccination, IFN-γ and TNF play critical roles in controlling parasite growth and/or survival, independent of macrophages and neutrophils, whereas IL-10 is important for controlling the vaccine-induced inflammatory response.

10.1128/mBio.02657-21.2 FIG S2 Role of IFN-γ and TNF in protective immunity induced by a 10^7^ P. yoelii 17X F3 + PHAD liposomal blood-stage vaccine. Groups of BALB/c mice (five mice/group) were immunized with three doses of the F3 + PHAD liposomal vaccine containing 10^7^ P. yoelii 17X pRBCs and challenged 1 month after the final vaccine dose with 10^5^ P. yoelii 17X pRBCs. A naïve control group that only received the challenge was also included. To examine the role of IFN-γ and TNF-α, mice received injections of rat Ig, anti-IFN-γ, TNF-α, or both IFN-γ and TNF-α antibodies on days −1, 1, 3, and 5 relative to challenge on day 0. (A) Parasitemias were monitored by blood films every second day following challenge. (B) Mice were monitored daily after challenge and euthanized if indicated by their clinical score or a weight loss of >15% postchallenge. (C) Hemoglobin levels were measured using a Hemocue201^+^ Analyser. The data are presented as means ± the SEM. +, Mice that were euthanized based on their clinical score or a weight loss of >15% postchallenge. Download FIG S2, TIF file, 0.2 MB. Copyright © 2021 Stanisic et al. 2021 Stanisic et al. https://creativecommons.org/licenses/by/4.0/ This content is distributed under the terms of the Creative Commons Attribution 4.0 International license.

### A lyophilized *P. yoelii* 17X liposomal vaccine has comparable protective efficacy to a freshly prepared vaccine.

Practical and logistic requirements, such as transport and storage, are important considerations for the eventual deployment of a vaccine in areas where malaria is endemic. Lyophilization is an approach that is used to ensure the long-term stability of liposomes. However, this process can alter the organization of the lipid bilayer and liposome size, and can affect immunogenicity (reviewed in reference 34). We lyophilized the vaccine and measured particle size. In fresh and lyophilized liposomes, particle sizes were 14.6 and 24.7 μm with spans (width of the size distribution) of 1.832 and 1.935, respectively. We then investigated the impact of lyophilization on vaccine immunogenicity and efficacy in BALB/c mice.

**FIG 10:**
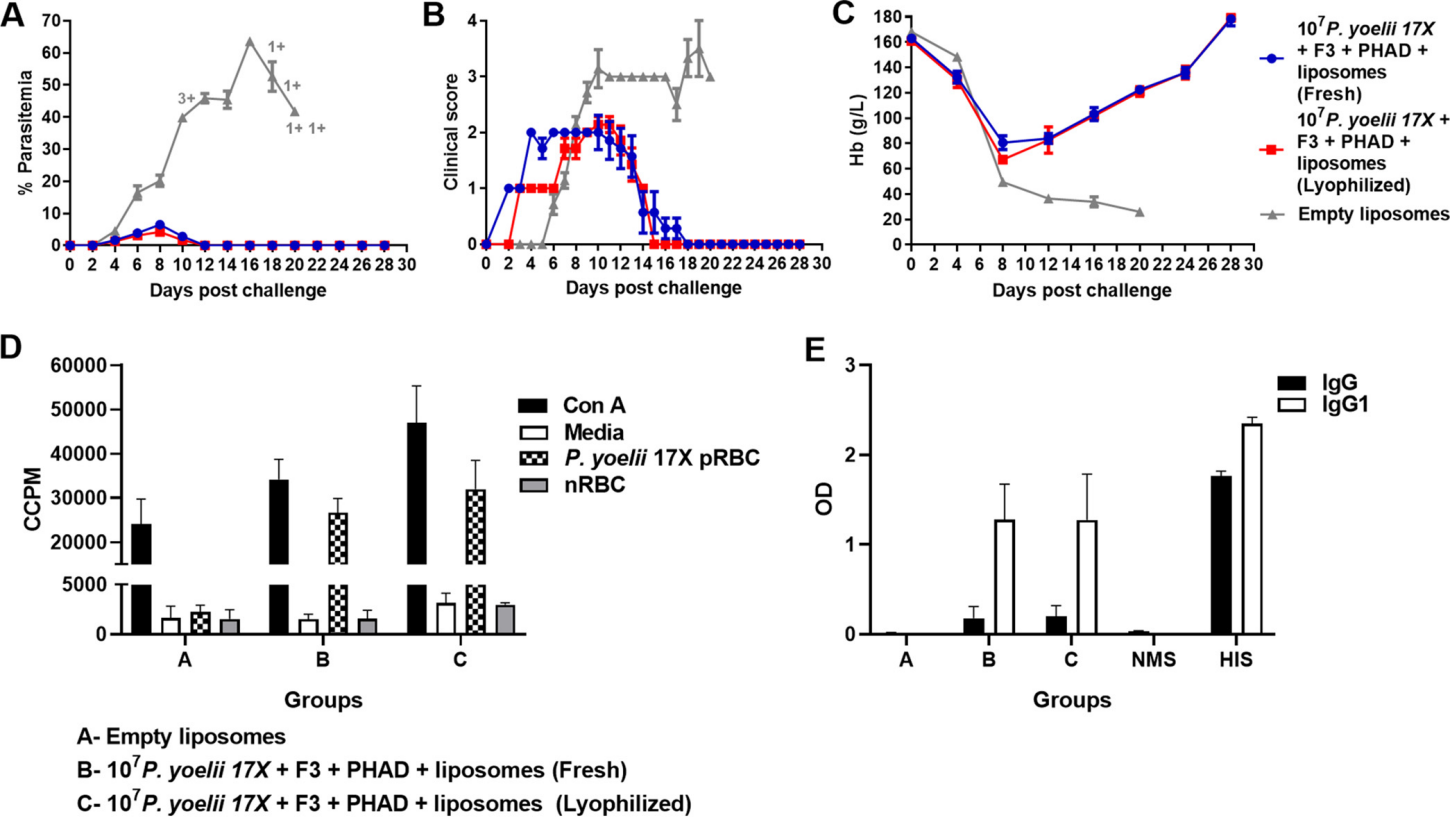
Immunogenicity and protective efficacy of lyophilized and freshly prepared 10^7^ P. yoelii 17X F3 + PHAD liposomal blood-stage vaccines. Groups of BALB/c mice (*n* = 7 mice/group) were immunized with three doses of a freshly prepared or lyophilized F3 + PHAD liposome vaccine containing 10^7^ P. yoelii 17X pRBCs and challenged 1 month after the final vaccine dose with 10^5^ P. yoelii 17X pRBCs. Control mice were immunized with empty liposomes. (A) Parasitemias were monitored by blood films every second day after challenge. (B) Mice were monitored daily after challenge and euthanized if indicated by their clinical score or a weight loss of >15% postchallenge. (C) Hemoglobin levels were measured using a Hemocue201^+^ Analyser. (D) Prechallenge splenocyte proliferative responses of vaccinated and control mice in response to fresh P. yoelii 17X pRBCs (*n* = 3/group). Proliferation was estimated by ^3^[H]thymidine incorporation and measured as corrected counts per minute (CCPM). Splenocytes from each mouse were tested with each stimulant in triplicate. (E) Prechallenge parasite-specific IgG and IgG1 responses in vaccinated and control mice (*n* = 7/group). Antibody to crude whole P. yoelii 17X parasite antigen was measured by ELISA. Serum was tested in duplicate at 1:100; samples from naive mice (NMS, normal mouse serum) and hyperimmune mice (HIS, hyperimmune serum) were included as negative and positive controls, respectively. The results are expressed as the optical density (OD) at 450 nm. The data are presented as means ± the SEM. +, Mice that were euthanized based on their clinical score.

Mean peak parasitemias were comparable between mice vaccinated with fresh and lyophilized formulations (Fig. 10A). Similarly, there were no significant differences between peak clinical scores or hemoglobin levels (Fig. 10B and C). Antibody and splenocyte proliferative responses did not differ significantly between mice vaccinated with either fresh or lyophilized vaccines (Fig. 10D and E). Significantly higher levels of IL-17A, TNF, and IFN-γ (all *P* < 0.05) were detected in the splenocyte culture supernatants derived from mice vaccinated with the lyophilized vaccine compared to the fresh vaccine (Table 1). Overall, the data demonstrated that despite some changes in the immunogenicity of the vaccine, lyophilized and fresh vaccines induced comparable levels of protective efficacy.

In a separate experiment, we also compared the efficacy of fresh and frozen vaccines in BALB/c mice. The frozen vaccine was minimally rehydrated prior to flash freezing on dry ice. After this, the sample was immediately thawed for administration. Overall, there were no significant differences in peak parasitemias, peak clinical scores, or hemoglobin when comparing the two groups that received the fresh and frozen P. yoelii 17X liposomal vaccines (see Fig. S3).

10.1128/mBio.02657-21.3 FIG S3 Protective efficacy of a frozen 10^7^ P. yoelii 17X F3 + PHAD liposomal blood-stage vaccine. Groups of BALB/c mice (five mice/group) were immunized with three doses of a fresh or frozen F3 + PHAD liposomal vaccine containing 10^7^ P. yoelii 17X pRBCs and challenged 1 month after the final vaccine dose with 10^5^ P. yoelii 17X pRBCs. (A) Parasitemias were monitored by blood films every second day after challenge. (B) Mice were monitored daily after challenge and euthanized if indicated by their clinical score or a weight loss >15% postchallenge. (C) Hemoglobin levels were measured using a Hemocue201^+^ Analyser. The data are presented as means ± the SEM. +, Mice that were euthanized based on their clinical score or a weight loss of >15% postchallenge. Download FIG S3, TIF file, 0.2 MB. Copyright © 2021 Stanisic et al. 2021 Stanisic et al. https://creativecommons.org/licenses/by/4.0/ This content is distributed under the terms of the Creative Commons Attribution 4.0 International license.

## DISCUSSION

A highly effective malaria vaccine is urgently needed to address the continued morbidity and mortality attributable to the malaria parasite. The renewed interest in whole-parasite malaria vaccines reflects a viable vaccine approach that addresses some of the limitations of subunit vaccines which have so far impacted their efficacy. Our previous work in rodent models of malaria demonstrated that whole parasite blood-stage vaccines can induce protection against blood-stage challenge in rodent models (19–22) and can induce parasite-specific strain- and species-transcending cellular immune responses in malaria-naive individuals (23). Here, we significantly extend this research by optimizing a blood-stage liposomal vaccine using the P. yoelii rodent malaria model and interrogating the immune response to identify CD4^+^ T cells, IFN-γ, TNF, and IL-10 as critical mediators of protective immunity. Importantly, we also demonstrate that lyophilization or freezing of the vaccine does not reduce its potency—an observation that will be critical to scaled vaccine manufacture and deployment.

A chemically attenuated blood-stage vaccine provides excellent protection in rodent models of malaria (19, 20), and a single vaccine dose induced parasite-specific immune responses in malaria-naive volunteers (23). Progressing this vaccine candidate, however, presents a number of significant challenges. First, the administration of red blood cells to humans, even those that are from a universal blood donor (blood group O RhD negative), presents a small risk of inducing antibodies against red blood cell antigens. Second, since rodent studies have shown that the parasites must be contained within intact red blood cells for the vaccine to induce protective immunity, the chemically attenuated blood-stage vaccine would require highly reproducible cryopreservation and thawing procedures, using specific equipment for this processing, to ensure administration of a precise dose. This is likely to be challenging in many areas where a malaria vaccine would need to be deployed. These issues can, however, be addressed by the liposomal vaccine approach.

The liposome is a well-recognized delivery system for drugs and antigens for vaccine development and can effectively replace the red blood cell to enable targeting and presentation of the parasite material to antigen-presenting cells. Red blood cell membranes could be removed prior to formulating the parasite material with the liposomes, and we have previously demonstrated that this can be achieved through the use of a novel immunomagnetic method (24). Liposomes are inherently unstable, with a limited shelf-life when they are in an aqueous dispersion. To facilitate long-term stability, lyophilization (freeze-drying) is used to remove water from the formulation; the addition of a cryoprotectant (e.g., trehalose) helps to stabilize the liposomes during this process (35). Reconstitution of a lyophilized vaccine just prior to administration would require minimal processing, particularly if the components are colyophilized in a single vial, as has recently been shown for a new thermostable RTS,S/AS01 vaccine formulation (36). A lyophilized vaccine formulation would have many advantages for deployment, particularly if thermostability is demonstrated. Disruptions to the cold chain occur for many reasons, and this can impact the efficacy and safety of vaccines that require uninterrupted storage at 4 to 8°C, resulting in increased costs and vaccine wastage if there are temperature excursions. Where vaccines can be reliably stored as a frozen product, freezing provides another option for storage without loss of potency. We have shown here that both a P. yoelii lyophilized vaccine formulation (stored for <24 h at 4°C prior to administration) and a P. yoelii frozen vaccine formulation (flash frozen and thawed without storage) retain their potency and efficacy. Further studies are now required to define the stability of the lyophilized and frozen vaccines by examining their vaccine efficacy following storage for different time periods and at different temperatures.

The P. yoelii 17X F3 + PHAD liposomal vaccine formulation was able to induce protection in both inbred and outbred mice. Vaccine efficacy was evaluated by measuring peripheral blood parasitemia, clinical scores, and hemoglobin levels in vaccinated mice following blood-stage challenge. Microscopically, the vaccinated, protected mice were free of parasites in their blood by the end of the postchallenge monitoring period. We cannot exclude the possibility that these mice continued to have a viable, submicroscopic infection beyond this monitoring period. This could be examined in future studies by collecting blood from vaccinated, protected mice at the end of the postchallenge monitoring period, transferring it into naive, recipient mice and monitoring the blood of the recipient mice for the development of a blood-stage parasitemia.

Both parasite-specific antibody and cellular responses were induced by the vaccine. In this study, only IgG and IgG subclasses were measured. Future studies could also examine the induction of other Ig classes, e.g., IgM and IgA, in vaccinated mice. Our data suggested that B cells were a necessary component of the protective immune response, since μMT mice were unable to control the parasite burden. However, these mice are known to have altered T-cell responses following infection. A recent study in Trypanosoma cruzi demonstrated a significant reduction in IFN-γ^+^ CD4^+^ T cells in the spleens and lymph nodes of μMT mice, compared to normal mice, following infection (27). Given the role that IFN-γ plays in immunity to the liposomal vaccine, it is possible the reduced immunity seen in μMT mice reflects a suboptimal IFN-γ response. If the significantly reduced immunity observed in μMT mice was due primarily to an absence of B cells, then it would be predicted that transfer of 1.5 ml of immune serum into naive recipients would transfer immunity, as has been shown in other models of malaria immunity (37). However, this did not occur, so we cannot exclude that vaccine-induced immunity is B cell independent. Further studies to address the potential contribution of a suboptimal IFN-γ response in vaccinated μMT mice may help to elucidate the true contribution of B cells to vaccine-induced immunity.

High levels of Th1 cytokines, e.g., parasite-specific IFN-γ, TNF, and IL-2, were detected in splenocyte culture supernatants, and we have previously shown that this combination of cytokines was the strongest correlation of immunity in mice immunized by a “controlled infection immunization” vaccine approach, where animals are given a controlled malaria infection under drug cover (21). The proinflammatory cytokines IFN-γ and TNF are important mediators of the adaptive immune response, and many studies have demonstrated their importance as key inflammatory cytokines for controlling parasite growth (reviewed in reference 28). Indeed, in this present study, antibody-mediated depletion of IFN-γ and TNF negatively impacted parasite control, and depletion of IFN-γ reduced the survival of vaccinated mice, indicating that they are critical components of the vaccine-induced protective immune response. IFN-γ is produced by CD4^+^, CD8^+^, and γδ T cells; depletion of the CD4^+^ T cells completely abrogated vaccine-induced protective immunity, and it is likely that they are one of the key sources of the inflammatory cytokines such as IFN-γ. TNF is produced by NK cells, CD4^+^ T cells, macrophages, monocytes, and neutrophils, and although there were higher parasitemias in mice depleted of TNF, this did not affect the survival of vaccinated mice. Depletion of macrophages and neutrophils individually did not affect vaccine-induced protective immunity; however, future studies could investigate the simultaneous depletion of both populations. Interestingly, high levels of the proinflammatory cytokine IL-17A were also detected; it is produced by Th17 cells, a unique CD4^+^ T-cell subset. The role of IL-17A in malaria infection, and thus an understanding of its potential role in vaccine-induced protective immunity, is still unclear; however, it has been shown to play a protective role in other protozoal infections (reviewed in reference 38). In field studies, an increase in IL-17-producing CD4^+^ T cells in peripheral blood was reported during P. vivax infection, although there was no correlation between Th17 cell numbers and morbidity or parasitemia (39). IL-17 has also been differentially associated with severe malarial anemia in residents of areas where malaria is endemic (40, 41). Although only low levels of anti-inflammatory IL-10 were produced, depletion of IL-10R in vaccinated mice led to an increase in clinical disease, highlighting the importance of immune regulation. Interestingly, cryopreservation of the vaccine formulation further augmented the production of IL-17A and the proinflammatory cytokines IFN-γ and TNF, and this was not accompanied by an increase in clinical disease compared to the fresh vaccine formulation.

A malaria vaccine that is able to provide protection against multiple strains and species of the malaria parasite circulating in an area where malaria is endemic would be advantageous. At a minimum, protective efficacy against multiple parasite strains should be the ultimate goal of any P. falciparum vaccine. We have previously shown in rodent models that different whole parasite blood-stage vaccines can induce strain- and species-transcending immunity, albeit at various levels depending on the parasite combinations used in the vaccine and challenge (19–21). Here, we show that the whole-parasite P. yoelii 17X blood-stage liposomal vaccine can provide significant cross-strain protection.

In summary, we show that immunization with a whole-parasite P. yoelii 17X F3 + PHAD liposomal vaccine induces both parasite-specific antibody and cellular immune responses. Protective immunity is critically dependent on CD4^+^ T cells, IFN-γ, and TNF and is not observed in μMT mice. Future work should include removal of the red blood cell membranes from the vaccine formulation using established protocols to determine whether there is any impact on the vaccine’s immunogenicity and protective efficacy (24). Importantly, we show that this liposomal vaccine approach is compatible with lyophilization or freezing without any loss of vaccine potency. This study supports continued development of this whole-parasite blood-stage vaccine and evaluation in a phase I clinical trial.

## MATERIALS AND METHODS

### Animals and ethics statement.

Six- to eight-week-old female BALB/c, C57BL/6, Swiss, and μMT mice were used. Inbred BALB/c, C57BL/6 and outbred Swiss mice were obtained from the Animal Resources Centre, Western Australia. μMT mice were originally obtained from the Jackson Laboratory and were maintained at the Griffith University Animal Facility. All animals were housed in the Institute for Glycomics Animal Facility under physical containment level 2 (PC2) conditions. All animal work was approved by the Griffith University Animal Ethics Committee under approval numbers GLY/15/17/AEC, GLY/7/17/AEC, and GLY/04/19/AEC.

### Rodent malaria parasites.

P. yoelii 17X, P. yoelii YM, P. chabaudi AS, and *P. vinckei* were initially obtained from Richard Carter (Edinburgh, UK) and were maintained by serial passage through inbred and outbred mice.

### Preparation of the vaccine.

Naïve mice were injected intravenously (i.v.) with P. yoelii pRBCs to generate parasites for the vaccines. Parasites were generated in the same mouse strain that the vaccine was intended for with the exception of μMT mice who received a vaccine containing parasites derived from their wild-type counterpart, C57BL/6 mice. To prepare the pRBCs for the vaccine, blood was collected from mice into a heparinized blood collection tube. The tube was centrifuged at 400 × *g* for 10 min, and the plasma was removed and discarded. The cell pellet was washed once with PBS and then resuspended in PBS for counting using a hemocytometer, and the parasitemia was determined using a Giemsa-stained thin blood film. The required number of pRBCs were aliquoted into Eppendorf tubes and placed at −80°C until required. The cell pellets, containing P. yoelii pRBCs, were subjected to six cycles of freezing and thawing prior to being formulated with liposomes.

Liposomes were prepared as previously described (22), using the thin film hydration method. They consisted of 1,2-dipalmitoylsn-glycero-3-phosphocholine (DPPC; Avanti Polar Lipids), dimethyldioctadecylammonium bromide (DDAB; Sigma-Aldrich) and cholesterol (Avanti Polar Lipids) in the ratio of 7:2:1. In addition, 3D(6-acyl) PHAD (PHAD; Avanti Polar Lipids) and a mannosylated core peptide, designated “F3” (22, 24), were also added (Fig. 1). F3 was synthesized as previously described (22). F3 was dissolved in methanol, and all other components were dissolved in chloroform. The solvent mixture was evaporated under vacuum to form a thin lipid film in the glass flask. The thin film was hydrated at 50 to 55°C with parasite antigen in PBS or in PBS alone (for empty liposomes) for 30 min, with the flask vortex mixed every few minutes. The vaccine was then ready for administration. Each vaccine dose contained 58.33 μg of DPPC, 16.66 μg of DDAB, 8.33 μg of cholesterol, 10 μg of F3, 25 μg of PHAD, and either the indicated number of P. yoelii 17X pRBCs or PBS alone (for the empty liposome vaccine). Naked liposomes did not contain F3 or PHAD.

For preparation of the frozen vaccine, the above procedure was followed except that the thin film was rehydrated at 50 to 55°C for 30 to 45 s, followed by vortexing for 15 s. This was continued up to 2 min 30 s until the thin lipid film was in solution. The contents of the flask were transferred to 5-ml Eppendorf tubes and flash frozen by incubating on dry ice for 1 h. Cryoprotectant was not added to this vaccine formulation prior to freezing. The vaccine was thawed at room temperature prior to administration.

For preparation of lyophilized liposomes, hydration of the thin lipid film was performed with PBS (20 mM [pH 7.0]) containing the cryoprotectant, trehalose (1.3 mM), and parasite antigen for 30 min, with the flask mixed every few minutes. The rehydrated liposomes were placed into glass vials, snap-frozen on dry ice-acetone mixture for 5 min, and then transferred to dry ice for an additional 5 min. The vials, with caps loosened, were placed in a freeze-dryer jar that was connected to the freeze-dryer (Christ Alpha 1-4 LOC) at −40°C and 0.1-mbar vacuum for 18 to 20 h. After removal from the freeze-dryer, the lyophilized liposomes were stored at 4°C until required for immunization later that day. Prior to immunization, the lyophilized liposomes were reconstituted in 1× Dulbecco-PBS. Particle size and size distribution of the liposomes were measured using a Mastersizer 3000 (Malvern Instruments, UK).

### Immunization and challenge of mice.

Mice were immunized subcutaneously on days 0, 14, and 28 with freshly prepared or lyophilized liposomes in a volume of 200 μl in PBS. Mice were challenged i.v. 1 month after the final vaccine dose with 1 × 10^5^ live pRBCs in a volume of 200 μl in PBS. Parasites were generated in naive mice of the same mouse strain being challenged with the exception of the μMT mice who received pRBCs derived from their wild-type counterpart, C57BL/6 mice. To prepare the pRBCs for challenge, blood was collected from mice into a heparinized blood collection tube. The tube was centrifuged at 400 × *g* for 10 min, and the plasma was removed and discarded. The cell pellet was washed once with PBS and then resuspended in PBS for counting using a hemocytometer; the parasitemia determined using a Giemsa-stained thin blood film. Postchallenge, mice were monitored every 2 days by Giemsa-stained thin blood film and every 4 days by measuring weight and hemoglobin (Hemocue201^+^ Analyser). Every day following challenge, the mice were also monitored using a clinical scoresheet. Mice that showed signs of severe distress, according to the clinical scoresheet or those that experienced >15% weight loss from the time of challenge, were euthanized using CO_2_ gas or by cervical dislocation. For experiments that required administration of monoclonal antibodies following challenge, experiments were blinded once the final dose of antibodies had been administered to address observer bias. For all other experiments, all postchallenge measurements were conducted blinded from the time of challenge.

### Splenocyte proliferation assay.

To examine the induction of parasite-specific cellular responses, splenocyte proliferation assays were undertaken. Spleens were removed from mice, placed into complete Roswell Park Memorial Institute medium (RPMI 1640, supplemented with 1% l-glutamine, 10% heat-inactivated newborn calf serum, 0.1% 2-mercaptoethanol, and 0.1% gentamicin) and were broken down manually using a 70-mm cell strainer, followed by two rounds of treatment with Geys lysis buffer to lyse the red blood cells. After each treatment, the splenocytes were washed with complete medium at 400 × *g* for 5 min at 4°C. Cells were counted using a hemocytometer and dispensed at 4 × 10^6^ cells/ml in complete medium (100 μl/well) into U-bottom 96-well plates. They were cultured for 72 h at 37°C and 5% CO_2_ in the presence of 100 μl of concanavalin A (10 μg/ml), complete medium, normal red blood cells (5 × 10^6^/ml), and live P. yoelii 17XL pRBCs (5 × 10^6^ pRBCs/ml) in triplicate. After 54 h, a portion of the culture supernatants were removed and placed at −80°C for soluble cytokine analysis. To assess proliferation, for the last 18 to 24 h of culture, 1 μCi of [^3^H]thymidine was added to each well. After the 72-h culture period, plates were frozen at −80°C. The plates were later thawed for harvesting onto glass fiber filter mats (Wallac, USA). ^3^[H]thymidine incorporation, expressed as corrected counts per minute (CCPM) was measured using a Microbeta2 microplate counter (Perkin-Elmer).

### Passive serum transfer studies.

Serum was collected and pooled from naive and 10^7^ P. yoelii 17X F3 + PHAD liposome-immunized BALB/c donor mice; for the latter, this commenced 2 weeks after the receiving the last vaccine dose. Naïve BALB/c recipient mice received 500 μl of the appropriate sera on days −1, 0, and 1 relative to the challenge with 1 × 10^5^ P. yoelii 17X pRBCs on day 0.

### ELISA for the detection of parasite-specific antibodies.

Indirect enzyme-linked immunosorbent assays (ELISAs) were undertaken to detect parasite-specific IgG. Ninety-six well flat-bottomed Maxisorp immunoplates (Nunc) were coated with 10 μg/ml of crude P. yoelii parasite lysate in bicarbonate coating buffer (pH 9.6) overnight at 4°C. Plates were blocked with 10% skim milk blocking buffer in PBS–0.05% Tween 20 for 1.5 h at 37°C. Plates were washed two times with wash buffer (0.05% Tween 20 in PBS) prior to adding prediluted serum from vaccinated mice. Plates were incubated for 1.5 h at 37°C and subsequently washed four to six times with wash buffer. Goat anti-mouse IgG-horseradish peroxidase (IgG-HRP; Invitrogen), IgG1-HRP, IgG2a-HRP, IgG2b-HRP, or IgG3-HRP (all Thermo Fisher Scientific) was added to the wells at 1:3,000, and the plates were incubated for 1.5 h at 37°C. Wells were washed six times with wash buffer, tetramethylbenzidine substrate (BD Biosciences) was added, and the plates were incubated for 10 min in the dark. The reaction was stopped using 1 M sulfuric acid, and the plates were read at 450 nm using a xMark microplate spectrophotometer.

### Cytometric bead array for the measurement of cytokines.

The detection of soluble cytokines in cell culture supernatants was undertaken using mouse Th1/Th2/Th17 and mouse inflammation CBA kits (BD Biosciences) according to the manufacturer’s instructions with minor modifications, as previously described (20). Culture supernatants from triplicate wells of the splenocyte proliferation assays were pooled and frozen at −80°C until used. Samples were acquired on a BD LSR Fortessa flow cytometer (BD Biosciences) and the data were analyzed using FCAP Array software version 3.0.1 (BD Biosciences).

### Depletion of cell populations and cytokines.

Following vaccination, mice were depleted of different cell populations and cytokines according to the following protocols. To assess levels of CD4^+^, CD8^+^, macrophage and neutrophil depletion, spleens removed from vaccinated, depleted, unchallenged mice were analyzed on a BD LSR Fortessa flow cytometer, as indicated below. Data were analyzed using FlowJo software version 10.6.2. In all experiments except for the macrophage depletion experiment, a control group of vaccinated mice received an equivalent amount of nonspecific rat Ig antibodies (Sigma-Aldrich) according to the same administration schedule.

To deplete CD4^+^ and CD8^+^ T cells, mice received intraperitoneal (i.p.) injections of 0.250 mg of anti-CD4^+^ (clone GK1.5; Bio X cell) or 0.250 mg of anti-CD8^+^ (clone 53-5.8; Bio X cell) antibodies on days −2, −1, 4, and 8 relative to the challenge on day 0. Depletion was confirmed by staining splenocytes on days 1, 9, and 16 postchallenge with CD3-V450 (clone 17A2), CD4-V500 (clone RM4-5), and CD8-PerCp-Cy5.5 (clone 53.6.7) (all antibodies from BD Biosciences).

To deplete macrophages, 100 to 200 μl of a liposome suspension (Liposoma, Amsterdam, Netherlands) containing clodronate (5 mg/ml) or PBS was administered i.v. on days −1 and 7 relative to the challenge on day 0. Depletion was confirmed by staining splenocytes on days 1 and 9 postchallenge with CD11c-FITC (clone HL3) and F4/80-PE (clone T45-2342) (both from BD Biosciences) and a Live/Dead stain (Invitrogen).

To deplete neutrophils, mice received i.p. injections of 0.5 mg of anti-Ly6G antibodies (clone 1A8; Bio X cell) on days −4, 1, 2, 5, 8, 11, and 14, relative to the challenge on day 0. Depletion was confirmed by staining splenocytes on days −3, 3, 6, and 9, relative to the challenge on day 0 with Ly-6G and Ly-6C-FITC (clone RB6-8C5) and CD11b-PerCp-Cy5.5 (clone M1/70) (both from BD Biosciences) and a Live/Dead stain (Invitrogen).

For depletion of cytokines, mice received 1 mg of anti-IFN-γ (clone XMG1.2; Bio X cell), anti-IL-10R (clone 1B1.3a, Bio X cell), or anti-IL-12p40 antibodies (clone C17.8; Bio X cell) i.p. on days −1, 0, and 1 relative to the challenge (14). In a second experiment, mice received 1 mg of anti-IFN-γ (clone XMG1.2; Bio X cell), anti-TNF (XT3.11; Bio X Cell), or both IFN-γ and TNF antibodies i.p. on days −1, 1, 3, and 5 relative to challenge.

### Statistics.

All statistical analyses were conducted using GraphPad Prism software version 8. All data are expressed as arithmetic means ± the standard error of the mean (SEM) unless stated otherwise. Data were analyzed using an unpaired *t* test to compare study groups and controls. A *P* value of <0.05 was considered significant for these analyses.
