# Supplementary figures and images for: Development and Evaluation of a Cryopreserved Whole-Parasite Vaccine in a Rodent Model of Blood-Stage Malaria
Source: mBio. 2021 Oct 19;12(5):e02657-21. doi: 10.1128/mBio.02657-21 (PMC8524336; doi:10.1128/mBio.02657-21)

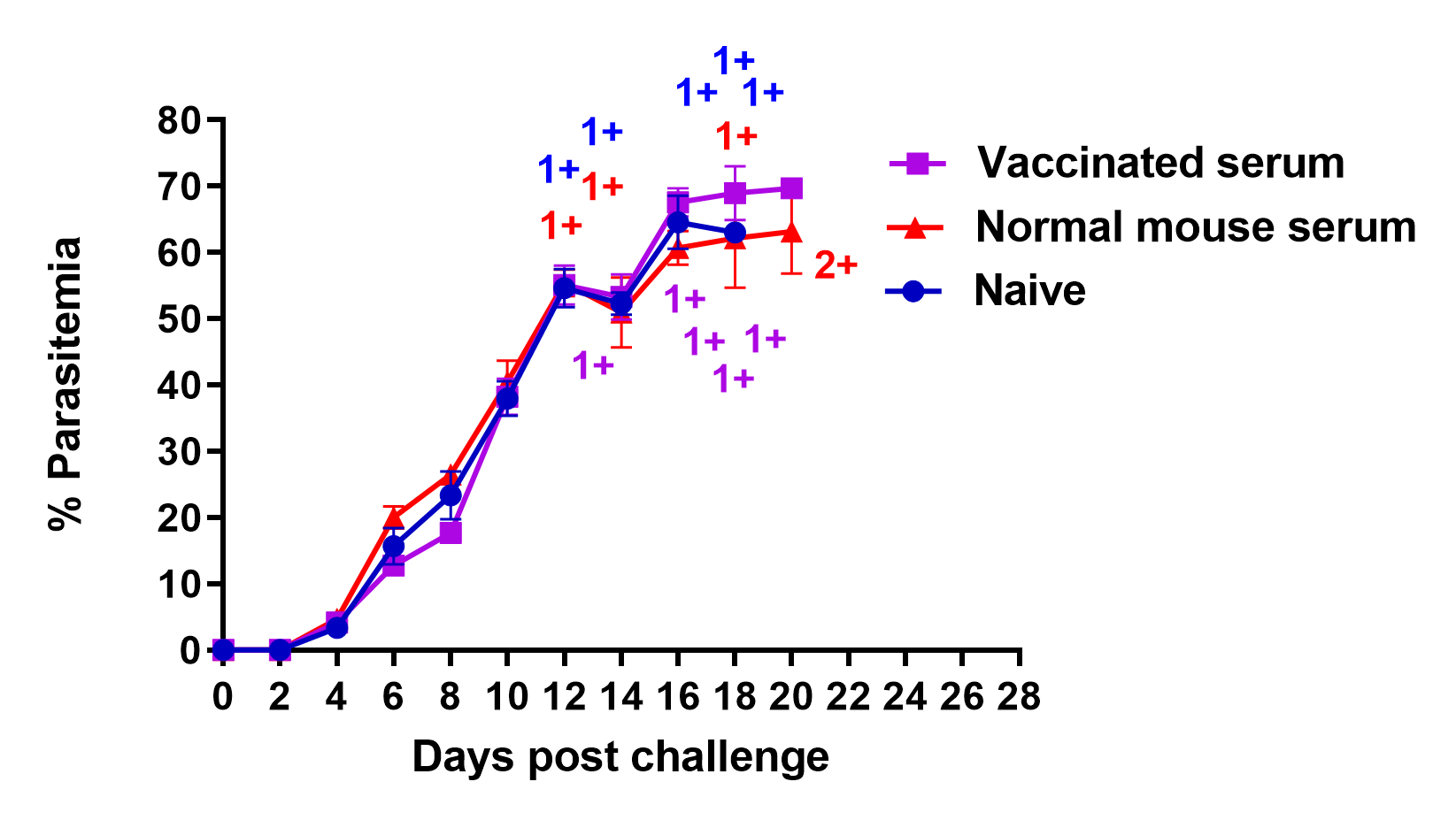

Supplement: FIG S1 [file mbio.02657-21-sf001.tif]

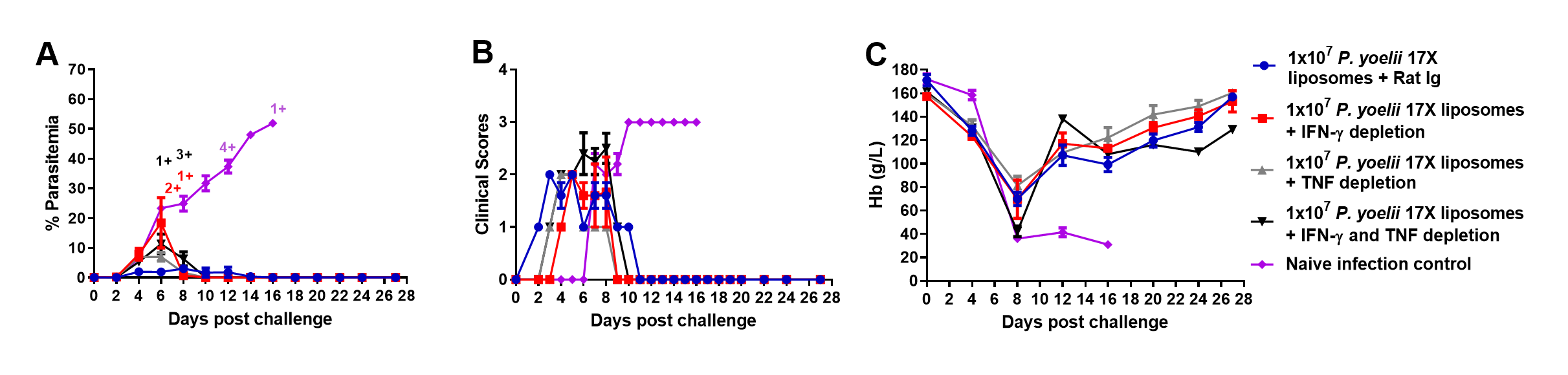

Supplement: FIG S2 [file mbio.02657-21-sf002.tif]

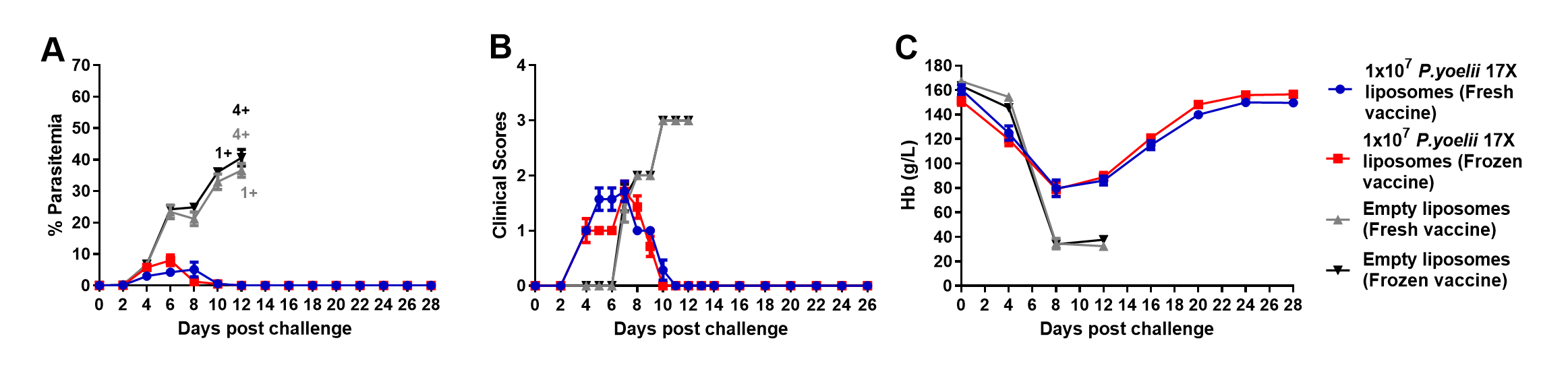

Supplement: FIG S3 [file mbio.02657-21-sf003.tif]
